# Structural, social, and safety barriers limiting mentorship participation among women in Kenya's animal health workforce: A cross-sectional survey of veterinarians and paraprofessionals

**DOI:** 10.14202/vetworld.2026.2915-2930

**Published:** 2026-07-11

**Authors:** James Mutiiria Kithuka, Timothy Muthui Wachira, Franklin Kebungo Ogwankwa, Joshua Orungo Onono

**Affiliations:** 1 Department of Public Health, Pharmacology and Toxicology, Faculty of Veterinary Medicine, University of Nairobi, P.O. Box 29053–00625 Kangemi, Nairobi, Kenya.; 2 Brooke East Africa, P.O. Box 43200–00100, Nairobi, Kenya.

**Keywords:** animal health workforce, career development, gender equity, Kenya, mentorship, professional advancement, veterinary professionals, women in leadership

## Abstract

**Background and Aim:**

Mentorship is essential for strengthening workforce capacity, leadership development, and career progression in veterinary and animal health professions. Despite increasing participation of women in Kenya’s animal health workforce, their engagement in structured mentorship programs remains constrained by multiple barriers. This study assessed the structural, social, and safety factors influencing mentorship participation among women veterinary and animal health professionals in Kenya and identified priorities for developing gender-responsive mentorship programs.

**Materials and Methods:**

A cross-sectional survey was conducted among 127 female veterinary and animal health professionals representing 24 of Kenya’s 47 counties. Participants were recruited using purposive and snowball sampling, and data were collected through a validated structured online questionnaire. Descriptive statistics summarized participant characteristics and perceived barriers, while Wilson 95% confidence intervals were calculated for key proportions. Pearson’s chi-square tests were used to examine associations between structural, social, and safety barriers and mentorship participation outcomes, with statistical significance set at p < 0.05.

**Results:**

Although 79.5% of respondents had previously received mentorship, 44.9% reported never or rarely receiving structured career guidance. The most common structural barriers were training costs (53.5%) and transport constraints (52.8%), whereas 60.6% frequently encountered stereotypes portraying animal health as a male profession. Professional competence was frequently questioned because of gender among 39.4% of participants, and 40.2% reported that safety or harassment concerns limited their participation in mentorship or professional development activities. Household and caregiving responsibilities also substantially restricted attendance at mentoring opportunities. Nine statistically significant associations were identified. The strongest relationship showed that combined exposure to safety concerns and gender stereotypes significantly increased withdrawal from professional opportunities (χ² = 19.30, p < 0.001). Career guidance was strongly associated with access to mentorship (χ² = 15.46, p < 0.001), whereas stereotype exposure was significantly associated with competence questioning, leadership exclusion, and professional withdrawal. Respondents prioritized flexible training schedules, financial and transport support, modular learning formats, safeguarding policies, and visible female role models to improve participation.

**Conclusion:**

Women's participation in mentorship within Kenya’s animal health workforce is constrained by interacting structural, social, household, and safety-related barriers. Developing gender-responsive mentorship programs that integrate financial support, flexible learning approaches, institutional safeguarding, and inclusive leadership policies will strengthen workforce equity, improve professional retention, and enhance leadership development across the animal health sector.

## INTRODUCTION

Livestock systems underpin food security, rural livelihoods, and economic development across sub-Saharan Africa. In Kenya, the livestock subsector contributes approximately 42% of agricultural gross domestic product (GDP) and 12% of the national GDP [1], highlighting the strategic importance of a competent, resilient, and well-distributed animal health workforce. Kenya's animal health workforce is regulated by the Kenya Veterinary Board under the Veterinary Surgeons and Veterinary Para-professionals Act 2011 and comprises approximately 7,000 actively retained professionals on the Kenya Veterinary Board register, including graduate veterinary surgeons, veterinary technologists, and veterinary technicians [2]. Women constitute an increasing proportion of this workforce, particularly among paraprofessionals and frontline personnel, although gender-disaggregated workforce data remain unavailable from the Kenya Veterinary Board [3, 4]. This trend reflects broader global patterns, with women accounting for approximately 80% of veterinary students in many high-income countries and increasing representation in low- and middle-income countries [5, 6]. Despite this demographic transition, women remain substantially underrepresented in leadership, decision-making, and senior advisory positions within veterinary organizations worldwide [6]. In Kenya, women occupy only 37% of leadership positions across public institutions, a disparity that also extends to the veterinary profession [7]. Recognizing these challenges, the World Organization for Animal Health (WOAH) Africa Continental Conference on Veterinary Workforce Development, held in Nairobi in November 2024, identified gender equality and women's professional advancement as strategic priorities for strengthening animal health systems across Africa [8].

Women employed in animal health systems continue to experience multiple structural, social, and occupational barriers that restrict their full participation in professional development. These barriers include unpaid caregiving responsibilities, mobility constraints, workplace gender bias, limited access to professional networks, and safety concerns during fieldwork and travel [9–11]. Among the available strategies for addressing these challenges, mentorship has consistently been recognized as an effective approach for promoting professional competence, leadership development, career progression, confidence, and workforce retention, particularly among women working in traditionally male-dominated professions [12, 13]. In the present study, a mentorship program is defined as a structured, institutionally supported relationship with explicit career development objectives, distinguishing it from informal mentoring relationships that may occur without predefined goals, duration, or institutional accountability [14]. Although professional organizations such as the Kenya Women Veterinary Association have established important networking opportunities for women in the veterinary profession [8], evidence on the barriers limiting women's participation in structured mentorship and career development programs remains limited. Furthermore, most published studies have focused primarily on women as livestock keepers, animal owners, or service recipients rather than as veterinary and animal health professionals navigating career advancement within the profession [15, 16].

Despite increasing recognition of gender equity as a priority within global animal health workforce development, important knowledge gaps remain regarding the specific barriers that limit women's participation in structured mentorship programs in Kenya. Existing literature has largely emphasized qualitative descriptions of gender inequality, leadership representation, or women's roles in livestock production, with little quantitative evidence examining how structural, social, household, and safety-related factors collectively influence mentorship participation among female veterinary and animal health professionals. Moreover, few studies have investigated the interactions among these barriers or identified factors associated with access to mentorship, career guidance, and professional advancement using quantitative analytical approaches. This lack of evidence limits the development of targeted, evidence-based, gender-responsive mentorship frameworks and institutional policies that can improve workforce retention, leadership development, and professional advancement for women in Kenya's animal health sector.

Therefore, this study was conducted to address these knowledge gaps by providing the first comprehensive quantitative assessment of mentorship barriers among women in Kenya's animal health workforce. Specifically, the study aimed to: (1) identify and quantify the structural, social, household, and safety-related barriers influencing women's participation in animal health mentorship programs; (2) examine factors statistically associated with mentorship access, career guidance, leadership opportunities, and professional participation; and (3) generate evidence to inform the design and implementation of gender-responsive mentorship frameworks and workforce development strategies that strengthen equity, professional retention, and women's leadership within Kenya's animal health sector.

## MATERIALS AND METHODS

### Ethical approval

Ethical approval for this study was obtained before participant recruitment from the University of Nairobi Biosafety, Animal Use and Ethics Committee (Reference No. BAUEC/2024/549) and the National Commission for Science, Technology and Innovation (Reference No. NACOSTI/P/24/36654). The study was conducted in accordance with the Kenya Data Protection Act 2019 and adhered to accepted ethical principles governing research involving human participants.

Electronic informed consent was obtained from all participants before access to the questionnaire. Participants confirmed their voluntary willingness to participate by selecting an affirmative consent statement at the beginning of the online survey. Participation was entirely voluntary, and respondents were free to discontinue the survey at any stage without penalty. No personally identifiable information was collected, and all study data were anonymized, securely stored, and reported only in aggregated form to maintain participant confidentiality and privacy.

### Study period and location

**Figure 1:**
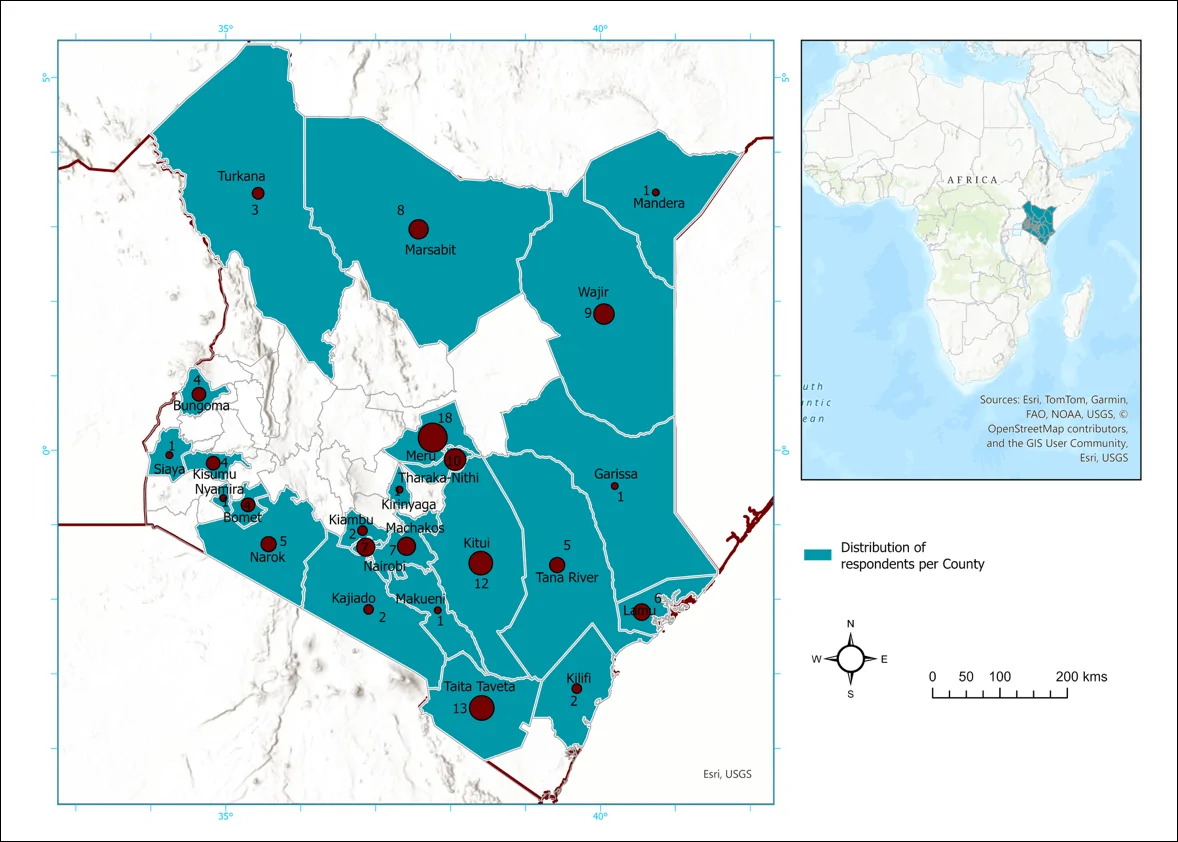
Geographic distribution of survey respondents across 24 Kenyan counties (n = 127). Counties are shaded according to respondent frequency, ranging from the highest participation in Meru (n = 18) to the lowest participation (one respondent each from Garissa, Kirinyaga, Makueni, Mandera, Nyamira, and Siaya). The map was generated using ArcMap Version 10.8.2 (Environmental Systems Research Institute [ESRI], Redlands, California, USA).

Data collection was conducted over a 2-month period from January to February 2026. During this period, eligible participants completed the online questionnaire through a secure electronic survey platform. The study was conducted throughout Kenya, representing diverse geographic, ecological, and socioeconomic regions. Responses were obtained from 127 participants residing in 24 of Kenya's 47 counties, providing broad national representation across the country's major administrative regions (Figure 1). Although participation was geographically diverse, the distribution of respondents varied among counties because recruitment relied on professional networking and snowball sampling.

### Study design

A cross-sectional survey was conducted to investigate the structural, social, household, and safety-related barriers influencing women's participation in animal health mentorship programs in Kenya. A cross-sectional design was considered appropriate because it enabled the simultaneous assessment of participant characteristics, mentorship experiences, and perceived barriers across a geographically diverse population during a single study period. The study focused on women actively engaged in animal health-related professions, including veterinary surgeons, veterinary technologists, veterinary technicians, animal health assistants, and agrovet practitioners.

### Study population and sampling

The study population comprised female animal health professionals aged ≥18 years who were actively practicing in Kenya during the study period. Eligible participants included veterinary surgeons, veterinary technologists, veterinary technicians, animal health assistants, and agrovet practitioners. Individuals who were male, younger than 18 years, not actively working in the animal health sector, or who submitted incomplete questionnaires were excluded from the study. All participants voluntarily provided informed consent before completing the questionnaire.

Participants were recruited using a non-probability purposive sampling strategy supplemented by snowball sampling. This approach was selected because no comprehensive gender-disaggregated national registry of women animal health professionals is currently available in Kenya, and the geographically dispersed nature of the target population necessitated network-based recruitment. Initial participants were identified through established professional networks, veterinary associations, training institutions, development organizations, and professional WhatsApp groups used by animal health practitioners. Seed participants were purposively selected to maximize geographic representation and professional diversity and were subsequently encouraged to refer eligible colleagues within their professional networks.

Recruitment was achieved across 24 of Kenya's 47 counties. The highest participation was recorded from Meru (n = 18, 14.2%), followed by Taita Taveta (n = 13, 10.2%) and Kitui (n = 12, 9.4%). This geographic concentration is acknowledged as a limitation of the sampling strategy. The questionnaire was administered electronically using Google Forms (Google LLC, Mountain View, California, USA). Because recruitment relied on snowball dissemination, the total number of individuals who received the survey link could not be determined; therefore, a formal response rate could not be calculated. Nevertheless, the final sample size of 127 participants is comparable with those reported in similar exploratory veterinary workforce studies [17].

### Sample size justification

Given the exploratory nature of this study and the absence of previous quantitative studies examining mentorship barriers among women animal health professionals in Kenya, no formal *a priori* sample size calculation was performed. The achieved sample size of 127 participants reflected the number of eligible respondents recruited during the study period using purposive and snowball sampling and is consistent with similar exploratory workforce investigations [17].

To evaluate the statistical adequacy of the sample, retrospective post hoc power analyses were performed using the observed chi-square statistics. Statistical power was estimated to be approximately 0.97 for the strongest association (χ² = 19.30, df = 1, α = 0.05) and 0.88 for the second strongest association (χ² = 15.46). The smallest statistically significant association (χ² = 5.81) had a moderate power of approximately 0.66; therefore, these findings should be interpreted with caution. In addition, certificate- and diploma-level respondents accounted for 68.5% of the study population, limiting the generalizability of the findings to graduate-level veterinarians and underscoring the need for cadre-specific investigations in future studies.

### Questionnaire development and data collection

Data were collected using a structured questionnaire developed specifically for this study, based on the published literature on mentorship, gender-related barriers, and professional development within animal health systems. The questionnaire consisted of eight thematic sections covering demographic and professional characteristics (Sections A-B), mentorship participation and barriers (Sections C-F), safety concerns (Section G), and institutional and policy factors (Section H). Section F incorporated a ranked-priority approach that required participants to identify and rank their three highest-priority support mechanisms, thereby allowing assessment of the relative importance of preferred interventions rather than simple binary selection.

Content validity was evaluated independently by three specialists with expertise in veterinary workforce development, gender in livestock systems, and survey methodology. The questionnaire was subsequently pilot-tested among eight women actively working in Kenya's animal health sector, after which three questions were revised to improve clarity and readability. No incentives were provided for participation. Internal consistency of all Likert-scale constructs was assessed prior to statistical analysis, with Cronbach's alpha exceeding the acceptable threshold of 0.70 for each construct. Participation was voluntary, and all questionnaire responses were collected anonymously.

### Data management and statistical analysis

Survey responses were exported from Google Forms to Microsoft Excel 2021 (Microsoft Corporation, Redmond, Washington, USA) for data cleaning and preparation. Data cleaning included verification of incomplete responses, assessment of internal consistency, and coding of categorical variables. Descriptive statistics were used to summarize respondent characteristics and study variables. For key proportions, 95% CIs were calculated using the Wilson score method.

Inferential statistical analyses were performed using IBM SPSS Statistics Version 26.0 (IBM Corp., Armonk, New York, USA). Pearson's chi-square tests were used to examine associations between selected categorical variables and mentorship participation outcomes. Ordinal variables were collapsed into binary categories to satisfy chi-square assumptions regarding minimum expected cell counts. Specifically, exposure to gender stereotypes was categorized as "often/always" versus "rarely/sometimes/never"; professional competence questioning was categorized as "often/very often" versus "never/rarely/sometimes"; and caregiving time was categorized as ≥4 hours/day versus <4 hours/day. A similar binary categorization was applied to the career guidance, household caregiving constraints, and leadership exclusion variables. A composite variable combining safety concerns (G1 = Yes) and frequent stereotype exposure (C2 = often/always) was created to evaluate their joint association with professional withdrawal. Statistical significance was established at p < 0.05 using two-tailed tests.

One respondent had missing data on training level (0.8%), and 10 respondents had missing data on program influence (7.9%). Missing values were managed using pairwise deletion without imputation because the proportion of missing data was low. Stratified analyses according to training level (paraprofessional versus graduate) were descriptive only. Formal chi-square analyses were not performed for the graduate subgroup (n = 39) because several contingency table cells failed to satisfy minimum expected frequency assumptions.

## RESULTS

### Respondent characteristics

The analysis included 127 female animal health professionals from 24 counties across Kenya. Diploma holders were the most common cadre (47, 37.0%; 95% confidence interval [CI]: 28.9-45.9%), followed by certificate holders (40, 31.5%; 95% CI: 23.7-40.3%), bachelor’s degree holders (34, 26.8%; 95% CI: 19.6-35.4%), and postgraduate qualification holders (5, 3.9%; 95% CI: 1.6-8.8%); one response had missing information on training level (0.8%). Together, certificate and diploma holders comprised 87 respondents, representing 68.5% (95% CI: 60.0-75.9%) of the sample, reflecting the predominantly paraprofessional and frontline composition of the workforce captured. The geographic distribution was broad, spanning 24 of Kenya’s 47 counties, with the highest participation from Meru (18, 14.2%), Taita Taveta (13, 10.2%), Kitui (12, 9.4%), Tharaka Nithi (10, 7.9%), and Wajir (9, 7.1%). Training level and geographic coverage are summarized in Table 1.

**Table 1:** Respondent characteristics, training level, and geographic distribution (n = 127).

**Variable**	**Category**	**n**	**%**	**95% Confidence interval**
Training level	Certificate	40	31.5	23.7-40.3
	Diploma	47	37.0	28.9-45.9
	Bachelor’s degree	34	26.8	19.6-35.4
	Postgraduate	5	3.9	1.6-8.8
	Missing	1	0.8	—
	Paraprofessional total (certificate + diploma)	87	68.5	60.0-75.9
Geographic distribution	Counties represented	24 of 47	—	—
	Meru (highest)	18	14.2	—
	Taita Taveta	13	10.2	—
	Kitui	12	9.4	—
	Tharaka Nithi	10	7.9	—
	Wajir	9	7.1	—
Household composition	Married/committed partnership	70	55.1	46.4-63.5
	Single parent	31	24.4	17.7-32.6
	Single, no children	19	15.0	9.7-22.4
	Pregnant/children under 5 years	20	15.7	10.3-23.3
	Caring for elderly/dependents	21	16.5	11.0-24.0
	Living with a disability	2	1.6	0.4-5.6
Decision-making autonomy	Decides independently	68	53.5	44.8-62.0
	Joint with family	29	22.8	16.3-30.9
	Employer/supervisor decides	29	22.8	16.3-30.9
	Family decides	1	0.8	—

**Regarding household composition, most respondents (70, 55.1%; 95% CI:** 46.4-63.5%) were married or in committed partnerships, 31 (24.4%) were single parents, and 19 (15.0%) were single with no children. Twenty respondents (15.7%) reported being pregnant or caring for children under 5 years of age, and 21 (16.5%) were caring for elderly or other dependents; two respondents (1.6%) reported living with a disability. Regarding decision-making autonomy for training attendance, 68 respondents (53.5%) reported making such decisions independently, 29 (22.8%) made them jointly with family members, and 29 (22.8%) reported that employers or supervisors made the decisions.

### Women’s representation and perceived reasons for underrepresentation

Perceptions of women’s representation were assessed using two survey questions. Overall, 112 participants (88.2%) indicated that women constituted a small minority in their professional environments; 14 (11.0%) estimated that women represented approximately half of their colleagues; and only one respondent (0.8%) indicated that women constituted almost all colleagues. When asked about the primary reasons fewer women enter animal health training and careers, the most frequently cited factors were cultural or gender norms (74, 58.3%), family or care responsibilities (53, 41.7%), safety concerns (44, 34.6%), limited role models (38, 29.9%), and limited access to information about opportunities (37, 29.1%). These findings establish the broader workforce context within which mentorship barriers operate.

### Mentorship participation and access to career guidance

**Overall, 101 of 127 participants (79.5%; 95% CI:** 71.7-85.6%) reported having previously had a mentor in animal health or a related field, whereas 26 (20.5%) had never had one. Among those with a mentor (n = 101), the most frequently cited effective mentor qualities were being technically knowledgeable (76, 75.2%), supportive and approachable (73, 72.3%), encouraging and motivating (70, 69.3%), respected in the profession (58, 57.4%), and a good communicator (57, 56.4%). Providing career guidance was cited by 55 respondents (54.5%), being ethical and professional by 55 (54.5%), being available and responsive by 49 (48.5%), and understanding gender-specific challenges by 47 (46.5%). Among the 26 respondents without a mentor, the most commonly cited barriers to accessing one were lack of professional networks (16, 61.5%), absence of female mentors (11, 42.3%), time constraints (8, 30.8%), and unwelcoming professional environments (6, 23.1%). These data are summarized in Table 2.

**Table 2:** Mentorship participation, effective mentor qualities, and barriers to mentor access (n = 127).

**Variable**	**Category**	**n**	**%**	**95% Confidence interval**
Mentorship participation	Ever had a mentor (Yes)	101	79.5	71.7-85.6
	Never had a mentor (No)	26	20.5	14.4-28.3
Structured career guidance	Yes (regularly)	50	39.4	31.3-48.1
	Occasionally	20	15.7	10.3-23.3
	No/Never	57	44.9	36.5-53.6
Preferred mentor gender	No preference	61	48.0	39.4-56.8
	Female	43	33.9	26.3-42.4
	Male	23	18.1	12.4-25.7
Effective mentor qualities among respondents with a mentor (n = 101)	Technically knowledgeable	76	75.2	65.7-82.8
	Supportive and approachable	73	72.3	62.6-80.5
	Encouraging and motivating	70	69.3	59.5-77.7
	Respected in the profession	58	57.4	47.4-66.9
	Good communication skills	57	56.4	46.4-66.0
	Provided career guidance/opportunities	55	54.5	44.5-64.2
	Ethical and professional	55	54.5	44.5-64.2
	Available and responsive	49	48.5	38.7-58.5
	Understands gender-specific challenges	47	46.5	36.8-56.5
Barriers to accessing a mentor among respondents without a mentor (n = 26)	Lack of professional networks	16	61.5	40.6-79.2
	No female mentors available	11	42.3	24.0-62.8
	Time constraints	8	30.8	15.2-51.9
	Unwelcoming professional environments	6	23.1	9.8-43.6

Access to structured career guidance on mentoring or leadership opportunities was less consistent. Fifty participants (39.4%; 95% CI: 31.3-48.1%) reported receiving such guidance, and 20 (15.7%) received it occasionally. In contrast, 54 participants (42.5%) reported not receiving guidance, and a further three (2.4%) had never received any, leaving 57 respondents (44.9%; 95% CI: 36.5-53.6%) with limited or no structured career guidance. Regarding mentor gender preference, 61 participants (48.0%) expressed no preference, 43 (33.9%) preferred a female mentor, and 23 (18.1%) preferred a male mentor.

### Structural barriers to mentorship participation

Structural barriers to participation were widespread. Overall, 94 of 127 participants (74.0%) indicated that structural barriers affected their participation at least moderately, including 58 (45.7%) reporting a moderate effect, 27 (21.3%) reporting a significant effect, and nine (7.1%) reporting a very significant effect. The remaining 33 participants (26.0%) indicated only a slight effect.

**Figure 2:**
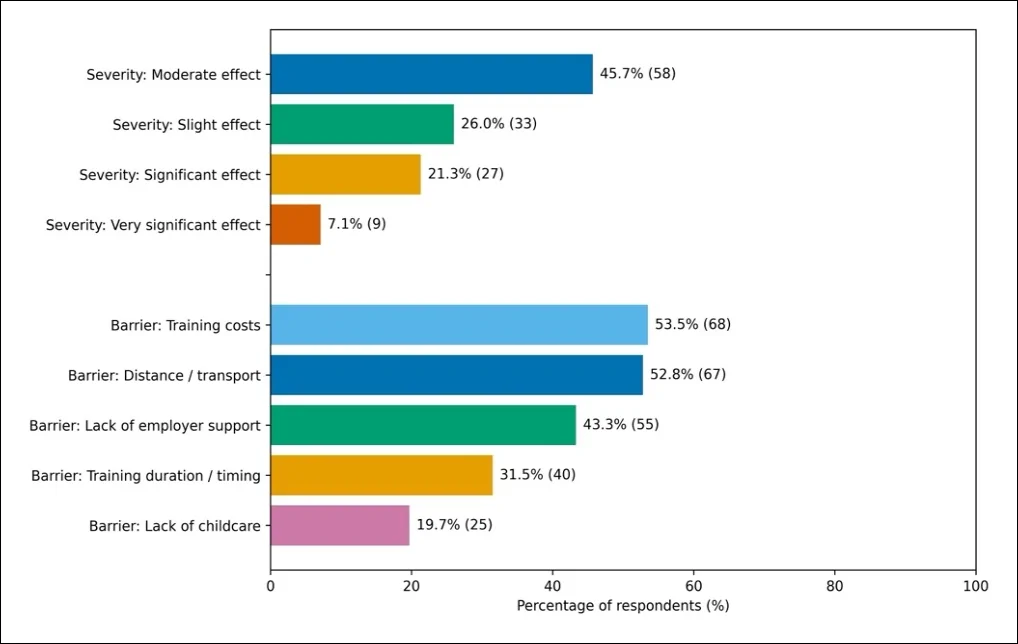
Distribution of structural barriers personally experienced by respondents, showing the proportion reporting each barrier type among 127 female animal health professionals in Kenya.

The most frequently reported personal barrier experiences were training costs (68, 53.5%; 95% CI: 44.9-62.0%), distance or transport constraints (67, 52.8%; 95% CI: 44.1-61.2%), family or care obligations (60, 47.2%), lack of employer support (55, 43.3%; 95% CI: 35.0-52.0%), training duration or timing (40, 31.5%), and lack of childcare facilities (25, 19.7%). Notably, the training cost barrier was reported at a substantially higher rate among paraprofessionals (53/87, 60.9%; 95% CI: 50.1-70.8%) than among graduate-level respondents (8/39, 20.5%; 95% CI: 10.6-35.3%), and this difference was statistically significant (χ² = 5.14, p = 0.023). The distribution of structural barriers is illustrated in Figure 2.

Regarding preferred structural improvements, participants were asked to rank their top-three priorities. Flexible scheduling received the highest weighted score (232) and was selected by 100 respondents (78.7%; 95% CI: 70.7-85.2%), followed by financial or transport support (weighted score = 186; 77 respondents, 60.6%), shorter or modular training formats (weighted score = 183; 78 respondents, 61.4%), training delivered closer to home (weighted score = 142; 70 respondents, 55.1%), remote or blended learning options (weighted score = 126; 59 respondents, 46.5%), and childcare support (weighted score = 62; 30 respondents, 23.6%). Structural barriers and preferred improvements are summarized in Table 3.

### Social and gender-related barriers

Social and gender-related barriers were widely perceived to influence professional participation. More than half of participants (70, 55.1%; 95% CI: 46.4-63.5%) agreed or strongly agreed that community or professional gender norms discouraged women’s engagement in animal health mentoring or leadership, whereas 21 (16.5%) were neutral and 36 (28.3%) disagreed or strongly disagreed.

**Views portraying animal health as a male profession were frequently encountered:** 77 participants (60.6%; 95% CI: 51.9-68.7%) reported hearing such views often or always, and a further 31 (24.4%) heard them sometimes; only 19 (15.0%) rarely or never encountered such views. Experiences of professional competence being questioned because of gender were also common: 50 participants (39.4%; 95% CI: 31.3-48.1%) reported this occurring often or very often, 33 (26.0%) sometimes, and 44 (34.6%) rarely or never. Exclusion from leadership or coordination roles because of gender was reported by 32 participants (25.2%; 95% CI: 18.5-33.4%), whereas 74 (58.3%) reported no such experience and 21 (16.5%) were uncertain.

Perceptions of client trust showed mixed patterns. Fifty-three respondents (41.7%) reported being trusted less than male colleagues, comprising those who felt slightly less trusted (36, 28.3%) and those who felt much less trusted (17, 13.4%). In contrast, 34 participants (26.8%) reported being equally trusted, 32 (25.2%) reported being more trusted than male colleagues, and eight (6.3%) were unsure. Gender-related barriers and trust perceptions are summarized in Table 4.

**Table 3:** Structural barriers to mentorship participation and preferred improvements (n = 127).

**Variable**	**Category**	**n**	**%**	**95% Confidence interval**
Barriers personally experienced (A5; multi-select)	Training costs	68	53.5	44.9-62.0
	Distance or transport constraints	67	52.8	44.1-61.2
	Family or care obligations	60	47.2	38.7-55.9
	Lack of employer support	55	43.3	35.0-52.0
	Training duration or timing	40	31.5	23.7-40.3
	Lack of childcare facilities	25	19.7	13.6-27.6
Training cost barrier by cadre	Paraprofessional (certificate/diploma) (n = 87)	53	60.9	50.1-70.8
	Graduate (bachelor’s/postgraduate) (n = 39)	8	20.5	10.6-35.3
Preferred structural improvements (A6; top-three ranked, weighted)	Flexible schedules (weighted score = 232)	100	78.7	70.7-85.2
	Financial/transport support (weighted score = 186)	77	60.6	51.9-68.7
	Shorter/modular training (weighted score = 183)	78	61.4	52.7-69.6
	Training closer to home (weighted score = 142)	70	55.1	46.4-63.5
	Remote or blended learning (weighted score = 126)	59	46.5	38.0-55.1
	Childcare support (weighted score = 62)	30	23.6	17.0-31.8

Weighted score = weighted priority score based on top-three ranking: first priority = 3 points, second priority = 2 points, and third priority = 1 point.

**Table 4:** Gender norms, stereotype exposure, professional trust, and leadership exclusion (n = 127).

**Variable**	**Category**	**n**	**%**	**95% Confidence interval**
Gender norms discourage participation (C1)	Strongly agree/Agree	70	55.1	46.4-63.5
	Neutral	21	16.5	11.0-24.0
	Disagree/Strongly disagree	36	28.3	21.2-36.8
Hearing that animal health is a male profession (C2)	Often/Always	77	60.6	51.9-68.7
	Sometimes	31	24.4	17.7-32.6
	Rarely/Never	19	15.0	9.7-22.4
Competence questioned because of gender (C3)	Very often/Often	50	39.4	31.3-48.1
	Sometimes	33	26.0	19.1-34.3
	Rarely/Never	44	34.6	26.8-43.2
Client trust compared with male colleagues (C4)	Much less trusted	17	13.4	8.5-20.5
	Slightly less trusted	36	28.3	21.2-36.8
	Equally trusted	34	26.8	19.8-35.0
	More trusted	32	25.2	18.5-33.4
	Not sure	8	6.3	3.2-12.0
Excluded from leadership roles (D3)	Yes	32	25.2	18.5-33.4
	No	74	58.3	49.5-66.6
	Not sure	21	16.5	11.0-24.0

### Household responsibilities and time constraints

**Figure 3:**
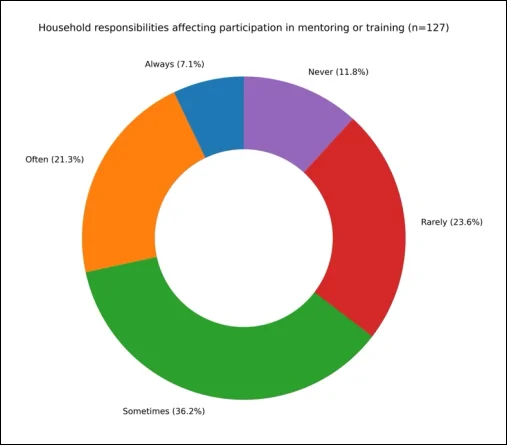
Frequency with which household and caregiving responsibilities prevented attendance at mentoring or training activities among respondents (n = 127), by response category.

Household and caregiving responsibilities represented a substantial competing demand alongside professional duties. Overall, 70 participants (55.1%; 95% CI: 46.4-63.5%) reported spending ≥4 hours daily on household or caregiving tasks, including 33 (26.0%) who spent more than 6 hours/day. When the response categories "sometimes," "often," and "always" were combined, 86 participants (67.7%; 95% CI: 59.2-75.2%) indicated that domestic responsibilities limited their attendance at mentoring or training activities at least occasionally; 36 of these respondents (28.3%) reported that such constraints applied often or always. The distribution of caregiving constraint frequency is presented in Figure 3.

### Safety concerns and professional engagement

Safety concerns were prevalent across the sample. Overall, 51 of 127 participants (40.2%; 95% CI: 32.0-48.9%) reported that safety or harassment concerns had limited their participation in mentorship or training opportunities, and 38 (29.9%; 95% CI: 22.6-38.4%) reported having declined or withdrawn from professional opportunities for safety-related reasons. The most frequently cited safety concern was unsafe travel at night (67, 52.8%; 95% CI: 44.1-61.2%), followed by intimidation or threats from clients or colleagues (49, 38.6%), fear of reputational damage (35, 27.6%), other safety concerns (24, 18.9%), harassment during training or professional events (23, 18.1%), and harassment during travel (9, 7.1%). Safety concern data are presented in Table 5.

**Table 5:** Safety concerns affecting professional participation (n = 127).

**Variable**	**Category**	**n**	**%**	**95% Confidence interval**
Safety/harassment limited participation (G1)	Yes	51	40.2	32.0-48.9
	No	76	59.8	51.1-68.0
Withdrew because of safety concerns (G3)	Yes	38	29.9	22.6-38.4
	No	89	70.1	61.6-77.4
Types of safety concerns (G2; multi-select)	Unsafe travel at night	67	52.8	44.1-61.2
	Intimidation/threats from clients or colleagues	49	38.6	30.6-47.3
	Fear of reputational damage	35	27.6	20.5-36.0
	Other safety concerns	24	18.9	13.0-26.7
	Harassment during training/events	23	18.1	12.4-25.7
	Harassment during travel	9	7.1	3.8-13.0

### Institutional and policy support

Institutional support for mentorship and professional development was variable. Lack of employer support was identified as a personal barrier by 55 participants (43.3%). Regarding influence over program design or priorities (D2), 69 respondents (54.3%, based on 117 valid responses after excluding 10 missing responses) reported significant or full influence, 21 (16.5%) reported some influence, and 27 (21.3%) reported very little influence. Organizational accountability for addressing gender barriers was affirmed by 74 participants (58.3%; agree or strongly agree).

The most frequently selected policy priorities (H3; multi-select) were capacity building, mentorship, and career development (99, 78.0%); gender-responsive policies and leadership representation (98, 77.2%); safe and inclusive work environments (96, 75.6%); flexible working conditions and caregiving support (93, 73.2%); and institutional culture change and awareness (66, 52.0%). Regarding equity indicators that organizations should monitor (H1; multi-select), respondents most frequently identified women enrolled in training (79, 62.2%), leadership roles held by women (77, 60.6%), income or career progression (77, 60.6%), confidence and satisfaction levels (73, 57.5%), safety incidents reported (51, 40.2%), and training completion rates (32, 25.2%).

Regarding preferred support mechanisms for increasing mentorship participation (F5; weighted ranking), financial or transport support received the highest weighted score (262) and was selected by 110 respondents (86.6%), followed by flexible learning options (weighted score = 216; 99, 78.0%), visible female role models (weighted score = 85; 44, 34.6%), clear safeguarding policies (weighted score = 84; 51, 40.2%), women-only mentoring spaces (weighted score = 69; 46, 36.2%), and peer-to-peer mentoring (weighted score = 46; 31, 24.4%). These findings are summarized in Table 6.

### Factors associated with mentorship and professional participation

Nine statistically significant chi-square associations were identified between selected barrier variables and participation outcomes (Table 7). The strongest association was the combined effect of safety concerns and gender stereotype exposure on withdrawal from professional opportunities (χ² = 19.30, df = 1, p < 0.001), with 40 respondents (31.5%) reporting both conditions concurrently.

**Table 6:** Institutional support, policy priorities, equity indicators, and preferred support mechanisms (n = 127).

**Variable**	**Category**	**n**	**%**
Employer support barrier	Lack of employer support reported	55	43.3
Influence over program design (D2)*	Significant/Full influence	69	54.3
	Some influence	21	16.5
	Very little influence	27	21.3
Organizational accountability (H2; agree/strongly agree)	Organizations accountable	74	58.3
Policy priorities (H3; multi-select)	Capacity building/mentorship/career development	99	78.0
	Gender-responsive policies/leadership	98	77.2
	Safe/inclusive work environments	96	75.6
	Flexible working/caregiving support	93	73.2
	Institutional culture change and awareness	66	52.0
Equity indicators to track (H1; multi-select)	Women enrolled in training	79	62.2
	Leadership roles held by women	77	60.6
	Income or career progression	77	60.6
	Confidence and satisfaction levels	73	57.5
	Safety incidents reported	51	40.2
	Training completion rates	32	25.2
Preferred support mechanisms (F5; weighted ranking)	Financial/transport support (weighted score = 262)	110	86.6
	Flexible learning options (weighted score = 216)	99	78.0
	Visible female role models (weighted score = 85)	44	34.6
	Clear safeguarding policies (weighted score = 84)	51	40.2
	Women-only mentoring spaces (weighted score = 69)	46	36.2
	Peer-to-peer mentoring (weighted score = 46)	31	24.4

*Percentages for D2 were calculated after excluding 10 missing responses (valid n = 117). Weighted score = weighted priority score.

**Career guidance was strongly associated with access to mentorship (χ² = 15.46, p < 0.001):** among respondents who had received career guidance (n = 50), 49 (98.0%) had previously had a mentor, compared with 52 of 77 (67.5%) among those who had not received guidance. Frequent exposure to gender-stereotyping views was significantly associated with professional competence being questioned (χ² = 14.34, p < 0.001) and, independently, with withdrawal from professional opportunities (χ² = 11.26, p = 0.001). Safety concerns were significantly associated with professional withdrawal (χ² = 13.34, p < 0.001), whereas frequent questioning of professional competence was associated with exclusion from leadership roles (χ² = 10.93, p = 0.001).

Caregiving-related associations were consistent across three variables. Respondents reporting ≥4 hours/day of caregiving were more likely to report that domestic responsibilities frequently prevented training attendance (χ² = 11.75, p = 0.001). Similarly, the presence of children under 5 years in the household (χ² = 6.82, p = 0.009) and caring for elderly or dependent household members (χ² = 5.81, p = 0.016) were each associated with more frequent participation constraints. All nine associations are presented in Table 7.

## DISCUSSION

### Principal findings and interpretation

This study is among the first to quantify the professional costs of gender-related barriers in the animal health workforce and provides the first Kenya-specific quantitative examination of how structural, social, household, safety-related, and institutional barriers constrain women’s participation in mentorship and professional development. The findings indicate that participation is shaped by interacting barriers rather than by isolated constraints. The strongest association identified was the combined influence of safety concerns and gender stereotype exposure on withdrawal from professional opportunities (χ² = 19.30, df = 1, p < 0.001), suggesting that these two barriers reinforce one another rather than operate independently. These findings position women’s limited participation in professional development as a systems-level challenge embedded in the design of training, mentorship, and career advancement pathways rather than as a reflection of individual motivation or capacity [9, 10].

### Informal mentoring and structured career guidance

Informal mentoring was common, but structured career guidance was inconsistent. The contrast between previous mentoring exposure (79.5%; 95% CI: 71.7-85.6%) and limited or no structured career guidance (44.9%; 95% CI: 36.5-53.6%) is one of the most programmatically important findings of this study. The association between career guidance and mentorship access (χ² = 15.46, p < 0.001) was among the strongest observed: among respondents who had received career guidance (n = 50), 49 (98.0%) had previously had a mentor, compared with 52 of 77 (67.5%) among those who had not received such guidance. This contrast is meaningful because informal mentoring relationships may provide encouragement and role modeling but may not consistently translate into structured professional sponsorship, access to leadership pathways, or career navigation support [12, 14]. The present findings suggest that Kenya’s animal health sector currently relies substantially on informal mentoring arrangements, which may lack explicit objectives, accountability mechanisms, and institutional linkages that characterize effective formal mentorship programs [10, 17]. This finding is consistent with Kipchirchir’s observation that workplace mentorship programs in Kenya contribute to gender diversity in leadership but remain unevenly implemented across cadres and institutions [17].

**Table 7:** Statistically significant associations between barriers and mentorship/professional participation outcomes (n = 127).

**Explanatory variable**	**Outcome variable**	**χ²**	**df**	**p-value**
Combined safety concerns and frequent stereotype exposure	Withdrawal from professional opportunities	19.30	1	<0.001
Career guidance on mentoring/leadership (Yes)	Access to mentorship (ever had a mentor)	15.46	1	<0.001
Frequent stereotype exposure (C2: often/always)	Competence questioned (C3: often/very often)	14.34	1	<0.001
Safety/harassment concerns limiting participation (G1 = Yes)	Withdrawal from professional opportunities (G3 = Yes)	13.34	1	<0.001
Household caregiving ≥4 hours/day (B2)	Care often/always limits attendance (B3)	11.75	1	0.001
Frequent stereotype exposure (C2: often/always)	Withdrawal from professional opportunities (G3 = Yes)	11.26	1	0.001
Frequent competence questioning (C3: often/very often)	Excluded from leadership roles (D3 = Yes)	10.93	1	0.001
Children under 5 years in household (B1)	Care often/always limits attendance (B3)	6.82	1	0.009
Elderly/dependent caregiving responsibilities (B1)	Care often/always limits attendance (B3)	5.81	1	0.016

All associations were statistically significant at p < 0.05 (two-tailed). Ordinal variables were collapsed into binary categories as described in Section 2.7. The combined safety-and-stereotype variable was constructed as both G1 = Yes and C2 = often/always (n = 40, 31.5% of the sample).

### Structural barriers and inequitable access to professional development

**Structural barriers, particularly training costs (53.5%; 95% CI:** 44.9-62.0%) and transport constraints (52.8%; 95% CI: 44.1-61.2%), were the most frequently reported personal barriers. The significantly higher rate of training cost barriers among paraprofessionals than among graduate-level respondents (χ² = 5.14, p = 0.023) indicates that structural barriers are not distributed uniformly across the workforce but are experienced more acutely by frontline cadres. This pattern is consistent with Food and Agriculture Organization analyses documenting logistical and financial barriers among women veterinary paraprofessionals in sub-Saharan Africa [15] and with Kenya-specific evidence from the contagious caprine pleuropneumonia vaccine value chain, where women encountered mobility, training access, and cost barriers despite demonstrated community demand for their services [16]. Comparable findings have also been reported among women participating in public health capacity development programs in Nigeria [18], suggesting that these structural constraints extend across professional contexts in the region. Respondents’ consistent prioritization of flexible schedules, financial and transport support, and shorter modular training reflects a practical demand for program adaptations that accommodate the realities of frontline practice.

### Gender stereotypes, professional credibility, and withdrawal

Gender stereotypes were a pervasive feature of respondents' professional lives. Frequent exposure to views portraying animal health as a male profession was reported by 60.6% of participants (95% CI: 51.9-68.7%). Stereotype exposure was significantly associated with the questioning of professional competence (χ² = 14.34, p < 0.001) and was independently associated with withdrawal from professional opportunities (χ² = 11.26, p = 0.001). Its combined effect, together with safety concerns, produced the strongest association in the study (χ² = 19.30, p < 0.001), indicating a synergistic rather than merely additive relationship between these two barrier domains. Begeny *et al.* [19] documented that gender bias persists even in professions where women are well represented numerically, whereas Kalbarczyk *et al.* [20] found that stereotype-driven questioning of competence can reduce women’s leadership aspirations and professional retention. In the present study, 41.7% of respondents reported being trusted less than male colleagues, illustrating how stereotypes may also manifest at the point of service delivery and potentially affect women’s professional reach, client relationships, and income. Kenya’s ranking of 75th out of 146 countries in the Global Gender Gap Index [21] and the finding that women hold only 37% of leadership roles in public institutions [22] provide the broader macro-structural context in which these professional-level patterns are situated.

### Cadre-specific implications for paraprofessionals

An important finding with intersectional implications was the predominance of certificate and diploma holders in the sample (68.5%; 95% CI: 60.0-75.9%). Unlike graduate-level professionals, paraprofessionals often occupy frontline roles with more limited access to formal professional networks, continuing education resources, institutional sponsorship, and structured career support. The Kenya Women Veterinary Paraprofessional Association, which was active at the WOAH Africa Continental Conference in Nairobi in November 2024 [8, 23], reflects organized professional agency at this cadre; however, the present data indicate that structural, social, and safety-related barriers continue to constrain progression even within this organized professional community. These findings suggest that attrition from professional development pathways in Kenya’s animal health workforce may begin at the paraprofessional-to-senior cadre transition rather than only at the graduate-to-leadership level. Cadre-differentiated interventions designed specifically to address the access, safety, and institutional constraints of frontline practitioners may therefore be more effective than generic gender policies applied uniformly across the workforce. Because this sample was predominantly paraprofessional, the findings should be interpreted with caution when generalizing to graduate veterinarians.

### Household responsibilities and time poverty

Household responsibilities and time constraints were consistent barriers across multiple measures. Respondents reporting ≥4 hours/day of caregiving were significantly more likely to report that domestic responsibilities frequently prevented attendance at training or mentoring activities (χ² = 11.75, p = 0.001). The presence of children under 5 years of age (χ² = 6.82, p = 0.009) and elderly or dependent caregiving responsibilities (χ² = 5.81, p = 0.016) were each independently associated with participation constraints. These findings are consistent with the concept of time poverty documented in Kenya’s health workforce leadership literature [9, 24], whereby the cumulative burden of unpaid domestic labor systematically reduces the time, energy, and decision-making autonomy available for professional development. An intersectional interpretation suggests that caregiving constraints do not operate uniformly. Women working in pastoralist counties or remote areas may face compounded disadvantages, in which geographic distance, limited professional networks, cultural expectations regarding women’s roles, and caregiving responsibilities interact in ways that standard residential training formats cannot adequately address. Kenya’s National Care Policy, which recognizes unpaid care work as a development priority [25], provides a policy entry point for interventions that could simultaneously address care burdens and support women’s professional participation.

### Safety as a workforce performance issue

**Safety concerns were among the most policy-relevant findings. The 40.2% (95% CI:** 32.0-48.9%) of respondents whose participation had been limited by safety or harassment concerns, the 29.9% (95% CI: 22.6-38.4%) who had declined or withdrawn from professional opportunities for safety reasons, and the strongest association in the study (χ² = 19.30 for combined safety and stereotype exposure) together underscore the severity and systemic nature of safety as a barrier. These findings are directly consistent with the WOAH regional recommendations on safe working environments for women in veterinary services [9] and with those of Eshikhena *et al.* [18], who documented safety barriers in public health professional development programs in Nigeria. Safety concerns in this context should be understood not only as gender equity concerns but also as animal health system performance issues because they affect whether women accept field assignments, attend mentorship activities, remain in professional development pathways, and progress toward senior roles that can improve veterinary service coverage across Kenya’s counties.

### Policy implications

The findings align with and extend several Kenyan and global policy frameworks. Kenya’s National Livestock Agenda 2025 calls for a strengthened and inclusive animal health workforce [1]; the current evidence quantifies the specific barriers that must be addressed to ensure this commitment equitably includes women in practice. The Veterinary Surgeons and Veterinary Para-professionals Act 2011 provides the regulatory framework within which the Kenya Veterinary Board can require gender-responsive professional development standards and gender-disaggregated data collection. The African Population and Health Research Center evidence brief on gender equality in health workforce leadership in Kenya [7] and the study by Saville *et al.* [9] on career pathways in Kenya’s health sector both indicate that structural and institutional change, not individual capacity building alone, is required to achieve gender-equitable career progression. The WOAH Africa Continental Conference on Veterinary Workforce Development, held in Nairobi in November 2024, explicitly called for systematic gender data collection and evidence-based workforce policies [8, 23]; this study contributes directly to that evidence base.

### Strengths of the study

This study has several noteworthy strengths. To the authors’ knowledge, it is the first quantitative survey to examine mentorship barriers specifically among women animal health professionals in Kenya, addressing a documented evidence gap and responding directly to calls from the WOAH 2024 Africa Continental Conference for systematic gender data in veterinary workforce planning [8, 23]. The multi-cadre, multi-county design, spanning 24 of Kenya’s 47 counties and capturing both veterinary surgeons and paraprofessionals, provides a breadth of professional experience rarely achieved in similar studies. The use of a context-specific validated questionnaire, with Cronbach’s α > 0.70 for all constructs, and the inclusion of a ranked-priority mechanism for support mechanisms enhance confidence in the reliability and practical relevance of the measures used. Beyond simple barrier identification, this study examined nine statistically significant associations with 95% CIs, providing a stronger evidentiary basis for programmatic recommendations than descriptive surveys alone. The construction of a composite safety-and-stereotype variable further represents a methodological contribution to the concurrent analysis of interacting barriers in workforce surveys.

### Limitations of the study

Several limitations should be considered when interpreting these findings. The cross-sectional design precludes causal inference; observed associations cannot establish directionality or rule out confounding. Snowball sampling through professional networks may have introduced selection bias and may have overrepresented digitally connected and network-active professionals while underrepresenting women in remote or underserved counties with limited internet access. The sample was dominated by certificate and diploma holders (68.5%), limiting generalizability to graduate-level veterinarians. Because the survey link was circulated through snowball sampling, the total number of individuals reached was unknown and a formal response rate could not be calculated. All data were self-reported and are therefore subject to recall and social desirability bias. The graduate subgroup (n = 39) was insufficient for reliable chi-square stratification, limiting formal cadre-comparative statistical analysis. Detailed dimensions of mentoring quality, including duration, frequency, structured objectives, and long-term career outcomes, were not captured systematically. Age and years of professional experience were not collected as continuous variables, limiting demographic stratification. The absence of a male comparison group limits direct assessment of gender-differential experiences. Despite these constraints, this study provides the first Kenya-specific empirical quantification of mentorship barriers in this workforce and offers a foundation for future longitudinal, mixed-methods, and gender-comparative research.

## CONCLUSION

This study provides the first quantitative evidence on mentorship barriers among women animal health professionals in Kenya and demonstrates that participation in mentorship and professional development is influenced by multiple interacting structural, social, household, safety-related, and institutional factors rather than by any single constraint. Training costs, transport challenges, caregiving responsibilities, gender stereotypes, and safety concerns were the most frequently reported barriers, while the strongest statistical association was the combined effect of safety concerns and exposure to gender stereotypes on withdrawal from professional opportunities (χ² = 19.30, p < 0.001). Structured career guidance was strongly associated with access to mentorship, underscoring the importance of formalized mentoring systems beyond informal professional relationships. The findings further indicate that paraprofessionals experience disproportionately greater structural barriers than graduate-level professionals, highlighting the need for cadre-specific approaches to professional development.

From a practical perspective, the findings support the development of gender-responsive mentorship programs that incorporate flexible training schedules, financial and transport assistance, remote or blended learning opportunities, childcare support, structured career guidance, safe working environments, and institutional accountability for gender equity. Strengthening these components could improve mentorship participation, workforce retention, leadership development, and ultimately the capacity of Kenya's animal health workforce to deliver equitable and effective veterinary services.

A major strength of this study is its multi-county, multi-cadre design and the use of a validated questionnaire, which enabled quantitative assessment of multiple interacting barriers and statistically significant associations. Nevertheless, the cross-sectional design, non-probability snowball sampling, predominance of paraprofessional respondents, reliance on self-reported data, and absence of a male comparison group limit causal inference and the generalizability of the findings to the entire veterinary workforce.

Future research should employ longitudinal and mixed-methods designs to evaluate how mentorship influences long-term career progression, leadership attainment, retention, and workforce performance. Comparative studies involving male professionals, graduate veterinarians, and other animal health cadres, together with intervention studies evaluating formal mentorship models and gender-responsive institutional policies, would further strengthen the evidence base for workforce planning.

Overall, the findings demonstrate that improving women's participation in mentorship requires coordinated structural and institutional reforms rather than individual-level interventions alone. Addressing financial, social, caregiving, and safety barriers through evidence-based, gender-responsive workforce policies will be essential for strengthening professional equity, enhancing leadership opportunities, and building a more resilient and inclusive animal health workforce in Kenya.

## DATA AVAILABILITY

The datasets generated and analyzed during this study are available from the corresponding author on reasonable request, subject to the provisions of the Kenya Data Protection Act 2019.

## GENERATIVE AI DECLARATION

The authors declare that no generative artificial intelligence or AI-assisted technologies were used in the writing, analysis, or preparation of this manuscript.

## AUTHORS’ CONTRIBUTIONS

JMK: Conceptualization, study design, methodology, data collection, data curation, formal analysis, interpretation of findings, and manuscript drafting. TMW: Study design, academic supervision, critical review, and manuscript revision for important intellectual content. FKO: Questionnaire development, data curation, formal analysis, interpretation of findings, and critical manuscript review. JOO: Study design, research supervision, critical manuscript revision for important intellectual content, and overall oversight. All authors have read and approved the final version of the manuscript.
